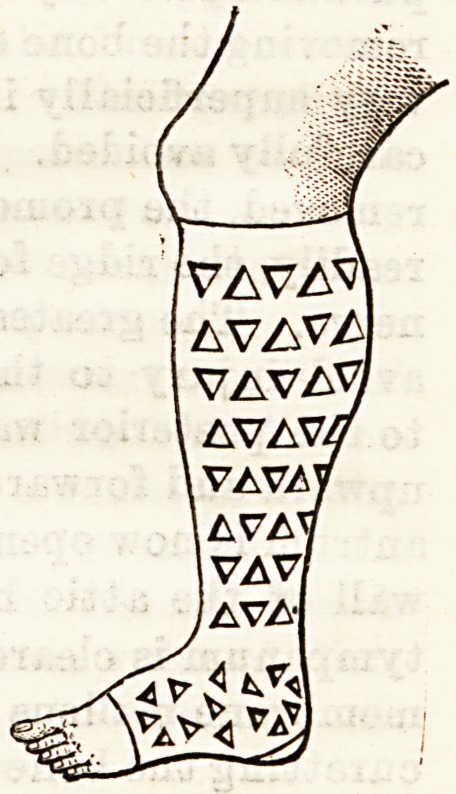# New Appliances and Things Medical

**Published:** 1896-07-04

**Authors:** 


### NEW APPLIANCES AND THINGS MEDICAL.

[We shall be glad to receive, at our Office, 428, Strand, London, W.C., from the manufacturers, specimens of all new preparations and appliances, which may be brought out from time to time.]

### THE "ALLENBURYS" FEEDER.

(Allen & Hanburys, Limited, Bethnal Green, E.)

Messrs. Allen and Hanbury have patented a new babies' feeding bottle which has several advantages over the old-fashioned one which has held sway in the nursery for so long. It has an opening at each end, and can therefore be cleaned with the greatest ease and thoroughness by holding under a tap of running water; and there are no angles or corners of any kind for the lodgment of food particles. There is no tube, and the nipple being of pure rubber can be turned inside out for cleansing. The valve stopper has a slit in the side which lets air in as fast as the food is sucked out; this prevents the nipple collapsing, and flatulence from swallowing air is obviated. The bottle is graduated in ounces to facilitate the accurate mixing of foods. The simplicity and consequent easy cleansing of the "Allenburys" feeder are undoubted advantages; its administration will need more care and attention than is the case with the tube feeding bottle, and this will be an advantage to baby, whose delicate digestion requires its meals to be carefully given as well as prepared, but will not, perhaps, suit the busy nurse or mother. This is not a fault in the feeder, however, and Messrs. Allen and Hanbury may well be congratulated on this latest addition to nursery appliances.

### THE "LATTICE" ELASTIC STOCKING.

We have subjected this stocking for varicose veins to a long practical test, and are satisfied that it possesses many advantages over the older varieties. It gives sufficient support to the veins without preventing the free flow of the column of blood; and, being less warm and allowing perspiration to escape, it is more comfortable to the wearer than any other artificial support we are acquainted with. It remains clean a long time, and when soiled it can be washed with much ease. There can hardly be a doubt as to its durableness. It is sold by the Lattice-Elastic Stocking Company, 70, Fenchurch Street, E.C.

### PERFUMED ANTISEPTIC FULLERS' EARTH.

(Arthur and Co., 69, Berners Street, W.)

Fullers' earth as a toilet preparation is too well known to require description, but as a dusting powder in certain skin diseases it is gradually becoming more and more appreciated. When applied to the skin it has the power of combining with the fatty secretions, and of thus rendering the skin cleaner from a surgical or medical standpoint. Its combination with an antiseptic such as boracic acid renders its action still more salutary. In the preparation of this powder pains have been taken to render it absolutely impalpable, and the absence of crystalline and irritating particles being thus secured, its value as an appplication to irritable surfaces is greatly enhanced.

## Figures and Tables

**Figure f1:**
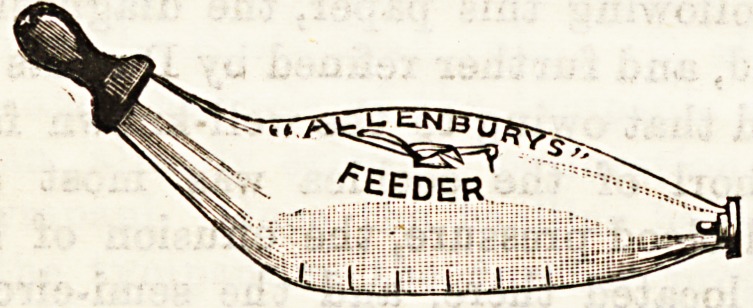


**Figure f2:**
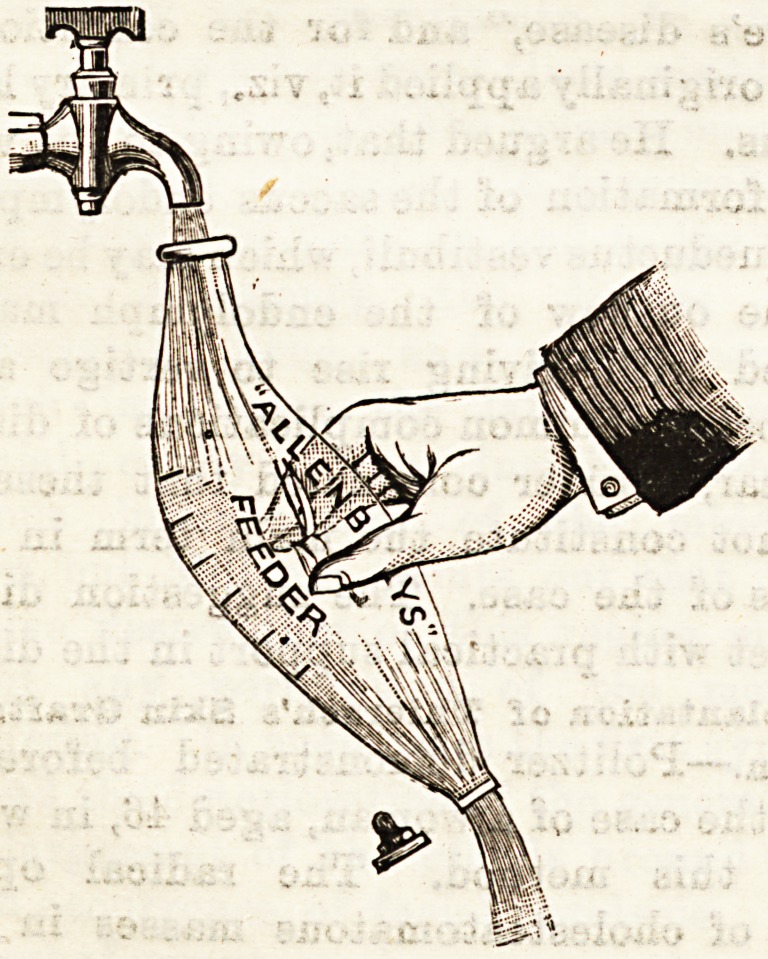


**Figure f3:**